# Editorial: Integrative pharmacological approaches for regenerating cartilage and bone tissue

**DOI:** 10.3389/fphar.2025.1613512

**Published:** 2025-04-30

**Authors:** Jiahui Tan, Jie Fang, Wen Liao, Lufei Wang

**Affiliations:** ^1^ Guangxi Key Laboratory of Oral and Maxillofacial Rehabilitation and Reconstruction & College and Hospital of Stomatology, Guangxi Medical University, Nanning, China; ^2^ State Key Laboratory of Oral Diseases and National Clinical Research Center for Oral Diseases, West China Hospital of Stomatology, Sichuan University, Chengdu, China

**Keywords:** bone regeneration, cartilage regeneration, pharmacological interventions, biomaterials, stem cell therapy

## Introduction

The destruction of cartilage and bone tissue—whether due to injury, degeneration, or disease—presents a significant clinical challenge that can severely impact mobility, function, and overall quality of life (Guo et al., 2023; Diaz-Solano et al., 2024). Conditions such as osteoarthritis (OA), osteoporosis, and fractures pose formidable obstacles due to the inherent limitations in the self-repair mechanisms of these tissues. Cartilage, being avascular, has a limited capacity for nutrient delivery and immune cell mobilization, which significantly diminishes its regenerative potential. While bone tissue is more metabolically active, its integration with surrounding tissues may be compromised by limited vascularization and slow healing.

To address these challenges, researchers have increasingly turned to integrative pharmacological approaches that include bioactive agents, growth factors, and stem cells to boost tissue regeneration (Adamička et al., 2021; Liao et al., 2024). These strategies aim to enhance healing by stimulating cellular activity, promoting repair, and improving tissue integration. Additionally, advances in tissue engineering and 3D printing are helping to create scaffolds and implants that support the regeneration of cartilage and bone (Gugliandolo et al., 2021; Wang et al., 2024). These developments offer promising avenues for more effective treatment options.

This Research Topic on “Integrative Pharmacological Approaches for Regenerating Cartilage and Bone Tissue” features 10 impactful studies, which consist of 4 original research articles, 3 critical reviews, 2 bibliometric analyses, and 1 meta-analysis, providing a comprehensive overview of diverse therapeutic strategies for regenerating cartilage or bone tissue. The contributions cover pharmacological interventions, innovative biomaterials, and advancements in stem cell therapy.

## Pathogenesis and treatment of osteoarthritis

Four studies within this topic focus on osteoarthritis (OA). Gao et al. demonstrated that age-related endoplasmic reticulum stress triggers YAP overexpression, which in turn leads to chondrocyte phenotype loss and OA progression. Their findings further revealed that treatment with pamrevlumab can alleviate these deleterious effects in cartilage-specific YAP overexpression transgenic mice, suggesting the therapeutic potential of targeting the endoplasmic reticulum stress–YAP–CTGF signaling pathway.

In another study, a bibliometric analysis conducted by Zhang et al. highlighted the growing importance of extracellular vesicles in OA research. The analysis revealed significant contributions from China, the United States of America, and Italy. The most frequently occurring keywords were “exosome,” “expression,” “knee OA,” “extracellular vesicle,” “mesenchymal stem cell,” and “inflammation.” These insights point toward the potential of extracellular vesicles as novel agents for OA treatment.

Liu et al. comprehensively summarized the roles of metal-organic frameworks (MOFs) as drug carriers and therapeutic agents in OA management and bone regeneration. The controlled drug release properties and tissue engineering capabilities of MOFs present long-term benefits, offering a new dimension in managing OA.

Hyaluronic acid (HA) intraarticular injection is a treatment used worldwide for knee OA. Tranexamic acid (TXA), a plasminogen activator inhibitor, inhibits fibrinolysis and matrix metalloproteases, which are key players in OA pathophysiology. According to Brochard et al., in a monosodium iodoacetate-induced murine model of knee OA, a single intraarticular injection of HA/TXA conjugate was shown to alleviate pain, slow OA progression, and provide chondroprotective effects—demonstrating its superiority over HA alone.

## Novel biomaterials for bone regeneration

Li et al. introduced a functionally graded bilayer membrane composed of poly (lactic-co-glycolic acid), nano-hydroxyapatite, and gelatin, developed by combining phase inversion with electrospinning techniques. This novel membrane not only provides enhanced barrier function and mechanical properties but also exhibits pronounced osteogenic bioactivity in both *in vitro* and *in vivo* models, offering fresh insights into barrier membrane fabrication for guided bone regeneration.

Another bibliometric study by Zhang et al. investigated research trends related to bioprinted hydrogels for bone regeneration. The findings reveal a rapidly growing field, with a strong emphasis on 3D printing technologies, scaffolds, and hydrogels. China emerged as the leader in terms of publication output, and future research appears to be oriented toward the utilization of gelatin, additive manufacturing techniques, and growth factors.

Yang et al. reviewed the properties of stimuli-responsive biomaterials, which can alter their mechanical characteristics, shape, or drug release profile in response to external or internal triggers. These materials have become increasingly significant in bone regeneration applications, representing a transformative approach to biomedical engineering that holds promise for future clinical innovations.

## Cell therapy in regenerative medicine

Liu et al. reviewed the effects of metformin—a widely used anti-diabetic drug—on mesenchymal stem cells (MSCs). The drug was found to promote MSC proliferation, differentiation, and resistance to aging, suggesting that metformin could enhance the therapeutic potential of MSCs in various regenerative contexts. This study highlights the pivotal role of cell therapy in the field of regenerative medicine.

In a comparative study, Li et al. evaluated MSCs derived from dental pulp, adipose tissue, placental amniotic membrane, and umbilical cord in an ovariectomy-induced mouse model of osteoporosis. The results indicated that dental pulp-derived MSCs not only maintained trabecular bone mass more efficiently but also exhibited superior immunoregulatory properties. These findings support the use of dental pulp-derived MSCs as the optimal cell source for cell therapy in postmenopausal osteoporosis.

Core decompression (CD) is a surgical operation commonly used for osteonecrosis of the femoral head (ONFH). Tang et al. contributed a meta-analysis that examined the combination of CD with regenerative therapies, such as bone marrow aspirate concentrate and bone-derived MSCs, in managing ONFH. Their analysis revealed that combining CD with regenerative approaches resulted in better outcomes for pain reduction and functional improvement compared to CD alone.

## Conclusion

This Research Topic of studies highlights emerging strategies in cartilage and bone regeneration, emphasizing novel biomaterials, stem cell therapies, and pharmacological interventions. These integrative approaches offer new avenues for improving therapeutic outcomes in osteoarthritis, osteoporosis, and bone defects, paving the way for future research and clinical applications. Strengthening interdisciplinary collaboration and clinical trials will be essential in translating these innovations into effective therapies, ultimately enhancing patient quality of life.
